# Supplementary material for: Management practices in hospitals: A public-private comparison
Source: PLoS One. 2023 Feb 24;18(2):e0282313. doi: 10.1371/journal.pone.0282313 (PMC9956577; doi:10.1371/journal.pone.0282313)
Supplement: S1 Appendix — (DOCX) [file pone.0282313.s001.docx]

${MS}_{ic}^{k}$ $X$ $\theta_{c})$ ${MS}_{ic}^{k}= \alpha_{c}+\beta_{c}{Public Hospital}_{ic}+{X'}_{ic}\delta_{c}+\theta_{c}+\varepsilon_{ic}$ $\beta_{c}$
**Supporting information: S1 Appendix**

**Table S1. Management Practice indicators**

| ***Performance monitoring*** |  |
| --- | --- |
| 1) Layout of patient flow | how well the patient pathway is configured at the infrastructure level and whether staff proactively improve their own work-place organisation |
| 2) Rationale for introducing standardization / pathway management | motivation and impetus behind changes to operations and what change story was communicated |
| 3) Standardisation and protocols | standardised procedures (e.g. integrated clinical pathways) that are applied and monitored systematically |
| 4) Performance tracking | performance is tracked using meaningful metrics and with appropriate regularity |
| 5) Performance review | performance is reviewed with appropriate frequency and communicated to staff |
| 6) Performance dialogue | quality of review conversations |
| ***Target setting*** |  |
| 7) Consequence management | differing levels of performance (NOT personal but plan/ process based) lead to different consequence |
| 8) Target balance | targets cover a sufficiently broad set of metrics |
| 9) Target interconnection | targets are tied to hospital objectives and how well they cascade down the organisation |
| 10) Target time horizon | whether hospital has a ‘3 horizons’ approach to planning and targets |
| 11) Targets are stretching | targets are appropriately difficult to achieve |
| 12) Performance clarity | easily understandable performance measures and performance openly communicated |
| ***Incentives*** |  |
| 13) Managing human capital | senior managers evaluated and held accountable for attracting, retaining, and developing talent throughout the organization |
| 14) Rewarding high performance | good performance is rewarded proportionately |
| 15) Removing poor performers | hospital is able to deal with underperformers |
| 16) Promoting high performers | promotion is performance based |
| 17) Attracting human capital | strength of the employee value proposition |
| 18) Retaining human capital | hospital will go out of its way to keep its top talent |

Note: The questionnaires and questions associated to each practice are available on the World Management Survey website (<https://worldmanagementsurvey.org/>). The data have been downloaded on the 20^th^ December 2019. See also Bloom and Van Reenen (2007).

**Table S2 - Management indicators: Descriptive statistics**

|  |  | mean | standard deviation | Min | Max |
| --- | --- | --- | --- | --- | --- |
| **Management Practice** |  | **2.70** | **0.53** | **1.20** | **4.35** |
|  | *Private* | 2.94 | 0.53 | 1.40 | 4.35 |
|  | *Public* | 2.59 | 0.49 | 1.20 | 4.20 |
| ***a) Performance monitoring*** |  | **2.92** | **0.65** | **1.00** | **4.80** |
|  | *Private* | 3.15 | 0.64 | 1.20 | 4.80 |
|  | *Public* | 2.82 | 0.63 | 1.00 | 4.60 |
| ***b) Target setting*** |  | **2.59** | **0.64** | **1.00** | **4.60** |
|  | *Private* | 2.80 | 0.66 | 1.00 | 4.60 |
|  | *Public* | 2.50 | 0.61 | 1.00 | 4.20 |
| ***c) Incentives*** |  | **2.47** | **0.63** | **1.00** | **4.33** |
|  | *Private* | 2.83 | 0.62 | 1.17 | 4.33 |
|  | *Public* | 2.32 | 0.58 | 1.00 | 4.17 |

Source: World Management Survey (Hospitals sub-sample)

| **Table S3 - Management Practices (management dimensions)** | | | | | |
| --- | --- | --- | --- | --- | --- |
|  | (1) | (2) | | (3) | (4) |
| VARIABLES | Management | Monitoring | | Targets | Incentives |
|  |  |  | |  |  |
| Public Hospital | -0.273*** | -0.269*** | | -0.258*** | -0.347*** |
|  | [0.034] | [0.045] | | [0.043] | [0.041] |
| Teaching Hospital | 0.006 | 0.011 | | 0. 017 | 0. 018 |
|  | [0.042] | [0.052] | | [0.053] | [0.048] |
| Hospital (medium size) | 0.201*** | 0.192*** | | 0.266*** | 0.158*** |
|  | [0.041] | [0.052] | | [0.052] | [0.048] |
| Hospital (large size) | 0.345*** | 0.359*** | | 0.474*** | 0.245*** |
|  | [0.044] | [0.057] | | [0.056] | [0.052] |
| country FE | yes | yes | | yes | yes |
| Observations | 1,058 | 1,058 | | 1,058 | 1,058 |
| R-squared | 0.262 | 0.183 | | 0.188 | 0.330 |
| Note: Robust standard errors in brackets | | | |  |  |
| *** p<0.01, ** p<0.05, * p<0.1 | | |  | | |

|  | \| **Table S3bis - Management Practices (management dimensions)** \| \| \| \| \| \| \| --- \| --- \| --- \| --- \| --- \| --- \| \|  \| (1) \| (2) \| \| (3) \| (4) \| \| VARIABLES \| Management \| Monitoring \| \| Targets \| Incentives \| \|  \|  \|  \| \|  \|  \| \| Public Hospital \| -0.302*** \| -0.298*** \| \| -0.293*** \| -0.370*** \| \|  \| [0.034] \| [0.045] \| \| [0.044] \| [0.041] \| \| Teaching Hospital \| -0.006 \| -0.004 \| \| 0. 017 \| 0. 009 \| \|  \| [0.041] \| [0.052] \| \| [0.053] \| [0.048] \| \| Hospital (log_n.beds) \| 0.128*** \| 0.139*** \| \| 0.169*** \| 0.088*** \| \|  \| [0.014] \| [0.017] \| \| [0.017] \| [0.016] \| \|  \|  \|  \| \|  \|  \| \| country FE \| yes \| yes \| \| yes \| yes \| \| Observations \| 1,058 \| 1,058 \| \| 1,058 \| 1,058 \| \| R-squared \| 0.284 \| 0.197 \| \| 0.210 \| 0.336 \| \| Note: Robust standard errors in brackets \| \| \| \|  \|  \| \| *** p<0.01, ** p<0.05, * p<0.1 \| \| \|  \| \| \|   **Table S4 - Management Practices (by country)** | | | | | | | |
| --- | --- | --- | --- | --- | --- | --- | --- | --- | --- | --- | --- | --- | --- | --- | --- | --- | --- | --- | --- | --- | --- | --- | --- | --- | --- | --- | --- | --- | --- | --- | --- | --- | --- | --- | --- | --- | --- | --- | --- | --- | --- | --- | --- | --- | --- | --- | --- | --- | --- | --- | --- | --- | --- | --- | --- | --- | --- | --- | --- | --- | --- | --- | --- | --- | --- | --- | --- | --- | --- | --- | --- | --- | --- | --- | --- | --- | --- | --- | --- | --- | --- | --- | --- | --- | --- | --- | --- | --- | --- | --- | --- | --- | --- | --- | --- | --- | --- | --- | --- | --- | --- | --- | --- | --- |
|  | | (1) | (2) | (3) | (4) | (5) | (6) | (7) |
| VARIABLES | | US | Canada | France | Germany | Italy | UK | Sweden |
|  | |  |  |  |  |  |  |  |
| Public Hospital | | -0.159** | -0.143 | -0.441** | -0.004 | -0.547*** | -0.249** | -0.659*** |
|  | | [0.062] | [0.352] | [0.185] | [0.069] | [0.101] | [0.130] | [0.113] |
| Teaching Hospital | | -0.034 | 0.075 | -0. 018 | 0. 003 | 0. 068 | -0. 043 | -0.145 |
|  | | [0.103] | [0.126] | [0.087] | [0.088] | [0.087] | [0.115] | [0.167] |
| Hospital (medium size) | | 0.299*** | 0.263*** | -0.223* | 0.653*** | 0.070 | 0.223** | 0.332* |
|  | | [0.071] | [0.076] | [0.118] | [0.070] | [0.202] | [0.104] | [0.185] |
| Hospital (large size) | | 0.457*** | 0.293*** | -0.059 | 0.830*** | 0.483*** | 0.097 | 0.069 |
|  | | [0.085] | [0.085] | [0.187] | [0.056] | [0.205] | [0.156] | [0.228] |
|  | |  |  |  |  |  |  |  |
| Observations | | 307 | 170 | 124 | 113 | 155 | 157 | 32 |
| R-squared | | 0.167 | 0.100 | 0.082 | 0.102 | 0.305 | 0.139 | 0.296 |
| Note: Robust standard errors in brackets | | | |  |  |  |  |  |
| *** p<0.01, ** p<0.05, * p<0.1 | | | |  |  | | | |

| **Table S5 - Management Practices: robustness** | | | | |
| --- | --- | --- | --- | --- |
|  | (1) | (2) | (3) | (4) |
| VARIABLES | baseline | with covariates | Propensity score ATE | Heckman selection |
|  |  |  |  |  |
| Public Hospital | -0.222*** | -0.258*** | -0.242*** | -0.263** |
|  | [0.033] | [0.032] | [0.036] | [0.107] |
| *σ* |  |  |  | 0.453 |
|  |  |  |  | [0.569] |
| country FE | yes | yes | yes | yes |
| propensity strata | no | no | yes | no |
| first stage | no | no | no | yes |
|  |  |  |  |  |
| Observations | 1,058 | 1,058 | 1,058 | 1,058 |
| R-squared | 0.273 | 0.182 | 0.189 | 0.337 |
| Note: Robust standard errors in brackets | | |  |  |
| *** p<0.01, ** p<0.05, * p<0.1 | | |  |  |
