# Supplementary material for: Management practices in hospitals: A public-private comparison
Source: PLoS One. 2023 Feb 24;18(2):e0282313. doi: 10.1371/journal.pone.0282313 (PMC9956577; doi:10.1371/journal.pone.0282313)
Supplement: Attachment — Submitted filename: Management Practices in Hospitals Lucifora.docx [file pone.0282313.s003.docx]

**Management Practices in Hospitals: A Public-Private Comparison by Claudio Lucifora**

Referee Report

The paper addresses a salient issue in health policy and management and has potential intriguing implications. It is also clearly written and easy to follow. However, it has several crucial weaknesses that need to be addressed by the author before becoming publishable. I will present them according to the structure of the paper.

Abstract

The first part of the abstract is not relevant as it is already known that management matters in emergency situation and the paper is based on rather old data. I suggest to radically change the first three lines of the abstract. I would openly state when data were collected as in the last decade there has been major changes in healthcare management in several countries, including Italy. Finally, I find the final part too simplistic and with no reference to the literature about how to improve management in the public sector. In this respect I suggest the author to get a bit more familiar with the public and healthcare management literature. The references show that he is not aware of a scholarly debate in the field of Public Administration and Public Management.

2. Management practices in hospitals

- The reference Chandra and Staiger (2007) appears unrelated to the statement made by the authors by the similar incentive structure in public and private hospital. Indeed, these two types of organizations tend to have different institutional goals; as it is well known private hospitals are mainly dominated by the search of shareholder value while public organizations are expected to pursue the public interest and they are, by definition, directly or indirectly, accountable to politics (see for example the Oxford Handbook on Public Management for an overview (Ferlie et al., 2005). Also, it is unclear how the survey and the authors treated non-profit hospitals, that are organizations that are generally owned by private entities but cannot share profits. It should specified this distinction and, if possible, it should be taken into account in the analysis.
- This sentence at the end of the section: “*It is important to stress that what is under investigation here is not the quality of healthcare or the services provided to patients, nor the resilience of hospitals to the coronavirus outbreak, but simply the management practices adopted in public and private hospitals, during normal times, to monitor performance, set targets, and recruit, retain and motivate the personnel. Is very important,”* is crucial. Particularly, it is essential that the author explains why he chose these three areas among all possible avoidable dimensions collected in the interviews, including some that are healthcare management specific.
- It should be reported when the data were collected given changes occurring in healthcare system worldwide
- It should reported (and justified) that the survey is based on interviews with clinicians and not managers *strictu senso.* As far as I understand, they acted as key informant about what happened in their hospitals. Obviously, they reported what perceived through the lenses of the clinical profession (see Numerato et al. 2012) to get a first idea about the relationship between management and medical doctors. It may be also useful to read a very recent paper that, by chance, investigated the same professionals in two Italian regions (Fattore et al, 2022).
- Please provide in the main text more info about the interview. If I remember well they are presented in one of the papers authored by Bloom.

3. Data and descriptive statistics

My preference is for papers that follow a traditional format: introduction, literature review (basically lacking in this paper), methods (including data and statistical methods), results, discussion and conclusions. It facilitates reading the paper.

- Present the statistical analysis of the difference in scores between public and private hospitals
- While Italy is missing in the figure presenting data country by country?
- What’s the meaning of quoting Syverson 2011? Doesn’t this paper refer to management in general? Anyway, it should be explained better why the parallel between the two papers matter

4. Main results

See my comment above about the structure of the paper. The regression model, with its various specifications, is the heart of the paper. The model, although simple, makes sense and it is clearly presented. Still needs some improvements (part in the methods section and part in the result section).

- I would avoid to use the expression management quality as the three indicators measure a part of what management is or can be
- I would avoid the expression management styles because it has a specific and narrower meaning in management studies
- Why the size of the hospitals is defined with two dummies (three classes). Don’t you have the stated size of the hospitals so to use it as a continuous variable? Please explain and justify
- This sentence should be moved in the discussion section where limitations of the study should be openly presented. “*Notice that, as previously discussed, some care should be used in interpreting the above results, since large hospitals may well have better procedures, but it could also be the case that better managers are more likely to be hired in larger organization. These different hypotheses, however, cannot be disentangled in our setting”*.

4.1 Robustness Check

I am not convinced by the Heckman model specification where the presence of competitor is used as IV. As rightly stated, the exclusion restriction assumption is unlikely to be respected. Why the level of competition is not simply used as control variable? In this respect, how competition is measured? Anyway, the part of the model presented in the discussion needs to presented earlier (in the method and results sections). More info are also needed to understand the variables to characterize hospitals; which are they? Are data reported by the key informants or collected from institutional sources (e.g., official eb sites)?

4.2 Discussion

This and the following sections are very scant with too few references and over simplifications. In the following I present some points of reflections.

- Larger hospitals are more difficult to be managed and thus require, in addition to others, the three elements considered by the authors. Smaller hospitals can perform well even with more basic management systems because informal relations, direct supervision by CEO and other top managers is easier, and organizational procedures and routines are more simple
- A large literature show that private and public management differences are substantial. Is it useful to use the same dimensions to measure management quality in the public and private sector? This was for sure the wish of the extreme New Public Management literature (see for example (Osborne and Gabler, 1992). For a critique to NPM see (Hood and Dixon, 2015). Maybe, looking for a co-author in the field of management and/or specifically in public management could make the difference, because she/he could put the empirical results of the study in the cultural context of disciplines that are debating these issues for decades.

Overall, this paper is potentially interesting and covers issues where there are relevant empirical gaps. However, as it stands is not publishable by PLOSE ONE. It needs a radical revision.

I do hope that my suggestions can help improve the paper.

Best

Giovanni Fattore

References

Fattore G, Numerato D, Salvatore D. Do Policies affect Management? Evidencce from a survey of clinicians in the Italian NHS. Health Service Management Research. 2022.

Ferlie E, Lynn L, Pollitt C edr. The Oxford Handbook of Public Management. Oxford University Press. 2007.

Hood C, Dixon R. A Government that worked better and costs less? Oxford University Press. 2015.

Numerato D, Salvatore D, Fattore G. The impact of management on medical professionalism: A review. Sociology of Health and Illness 2012; 34 (4): 626-644.

Osborne D, Gaebler T. Reinventing Government. New York. Penguin Press. 1992
