# Supplementary material for: Management practices in hospitals: A public-private comparison
Source: PLoS One. 2023 Feb 24;18(2):e0282313. doi: 10.1371/journal.pone.0282313 (PMC9956577; doi:10.1371/journal.pone.0282313)
Supplement: Attachment — Submitted filename: Response to Reviewers_PlosOne_2023.docx [file pone.0282313.s005.docx]

**Rebuttal letter**

**Response to Editor’s comments**

*1. reviewer 2 raises again some concerns about your methodology and, in particular, the lack of use of some suggested control variables. I read carefully the justifications by the author and some of them convinced me to assign minor revisions. However, I suggest an effort in order to use at least the typology of hospitals (mainly the difference between teaching and research hospitals and other hospitals) as a control variable. It may be really interesting..*

ANSWER: I agree with you and the referee that including hospital type as additional control variable may prove interesting for the empirical analysis. To meet both your and the referee’s concerns, I applied to the WMS initiative asking for information on “hospital type” which was not available in the public-use database. Luckily I was able to obtain the requested info. The paper has therefore been amended accordingly. In the revised version, I discuss the implications of hospital type for public-private differences in management styles. Next, I re-estimate all the specifications including a dummy proxying for “hospital type” (research/teaching versus other).

A few typos and references have been also amended.

***Reviewers' comments:***

**Reviewer #1**: All comments have been addressed

**Reviewer #2:** 

*Overall, I think that the structure of the paper and its theoretical positioning is improved as the author(s) have addressed most of my suggestions. Indeed, most of efforts have been done in terms of improvement of the assessment of prior studies on the topic. However, I think that the paper is still lacking in what concerns the methodology as the regression models still suffer of the use of more control variables. This can prevent the strength and the generalizability of the results.*

*In the previous review I suggested the authors to include more control variables such as (i) type of hospitals (general hospitals, teaching hospitals, research hospitals); (ii) hospital performance (e.g. quality and efficiency) (as it might possible that the adoption of management practices is more likely to occur within better performers); (iii) characteristics of served population; (iv) hospital complexity. Indeed, in the previous draft of the paper, the authors only used the hospital size as control variable. However, in this new version of the paper, hospital size and country dummies remain the only control variables. The authors answered to the comment that they have discussed the possible implication of omitting these variables. I think that the results in this version could be not strong and generalizable.*

*On the basis of this, I would suggest again the authors to enrich the regression model in order to make their research more robust. I think that the article is interesting and with a good potential, but this issue can strongly prevent the contribution of the paper to the literature.*

ANSWER: I agree with the referee that including additional variable to control for hospital type may prove interesting for the empirical analysis. Unfortunately, such information was not available in the WMS public-use database, and in my previous revision I could not address the referee’s request to add further control variables to the empirical analysis. However, given that the referee considers the “type” of hospital as a serious confounding factor, I approached the WMS initiative and asked for the additional information on hospital type to be provided. Luckily, the information on hospital teaching status was available in the original questionnaire and my request was approved by the WMS initiative. The revised version of the paper, which I am herein submitting, contains an additional variable capturing whether the hospital is a “research/teaching” versus other. Unfortunately, no information on hospital performance was asked in the survey. In practice the paper has been thoroughly revised as follows: first, a discussion of the implications of hospital type for the analysis has been added in the Introduction and in the data sections; second, the empirical analysis has been amended to include a dummy for teaching hospital status in all estimated specifications. The tables and figures have all be revised accordingly. A few typos and references have been also amended.
